# First Chemical Investigation of Korean Wild Mushroom, *Amanita hemibapha* subsp. *javanica* and the Identification of Anti-*Helicobacter pylori* Compounds

**DOI:** 10.3390/ph15020152

**Published:** 2022-01-27

**Authors:** Seulah Lee, Akida Alishir, Tae Wan Kim, Dong-Min Kang, Rhim Ryoo, Changhyun Pang, Mi-Jeong Ahn, Ki Hyun Kim

**Affiliations:** 1 School of Pharmacy, Sungkyunkwan University, Suwon 16419, Korea; seulah@kopri.re.kr (S.L.); akida.alishir@gmail.com (A.A.); asde8282@naver.com (T.W.K.); 2 Division of Life Sciences, Korea Polar Research Institute, KIOST, Incheon 21990, Korea; 3 College of Pharmacy and Research Institute of Pharmaceutical Sciences, Gyeongsang National University, Jinju 52828, Korea; kdm7105@gnu.ac.kr (D.-M.K.); amj5812@gnu.ac.kr (M.-J.A.); 4 Special Forest Products Division, Forest Bioresources Department, National Institute of Forest Science, Suwon 16631, Korea; rryoo@korea.kr; 5 School of Chemical Engineering, Sungkyunkwan University, Suwon 16419, Korea; chpang@skku.edu

**Keywords:** *Amanita hemibapha* subsp. *javanica*, Amanitaceae, fatty acid derivatives, steroids, LC/MS analysis, CEA, anti-*H*. *pylori* activity

## Abstract

*Amanita hemibapha* subsp. *javanica* (Amanitaceae) is an edible Korean wild mushroom. *A. hemibapha* subsp. *javanica* is often confused with *A. subjunquillea*, known as the East Asian death cap, which is potentially fatal when ingested. This study aimed to conduct the first chemical investigation of *A. hemibapha* subsp. *javanica*, which resulted in the isolation of seven fatty acid derivatives (**1**–**7**) and three steroids (**8**–**10**) from the MeOH extract of its fruiting bodies, and their structures were determined by comparing their NMR spectroscopic data with those previously reported, along with the data from LC/MS. Compound **1** was reported previously without the identification of its absolute configuration; its structure, including the absolute configuration was confirmed for the first time, in this study, by using ^1^H NMR and its fragmentation patterns in MS/MS data, and LC/MS analysis. A recently developed method using competing enantioselective acylation (CEA) coupled with LC/MS analysis was applied for determining the absolute configuration of compound **1**, which revealed the 11*S*-configuration. In the anti-*Helicobacter pylori* activity test, compound **3** showed antibacterial activity against *H. pylori* strain 51 with 38.0% inhibition, comparable to that of quercetin (34.4% inhibition) as a positive control. Specifically, compound **4** displayed the most potent antibacterial activity against *H. pylori* strain 51 with 80.5% inhibition at the final concentration of 100 μm with a MIC_50_ value of 72 μm. These findings suggested that the active compound **4** is a natural antibiotic that may be used in the development of novel antibiotics against *H. pylori.* In addition, the first chemical investigation of *A. hemibapha* subsp. *javanica* revealed that this mushroom can serve as a promising natural source for the bioactive natural products.

## 1. Introduction

Mushrooms in the genus *Amanita* are the most well-known psychoactive and poisonous basidiomycete fungi [1]. The *Amanita* genus consists of cyclopeptide-containing mushrooms, which are responsible for over 90% of all fatal mushroom poisoning instances [2]. The major species that contain cyclopeptide toxins such as α-amanitin, β-amanitin, phalloidin, and phallacidin include *A. phalloides*, *A. virosa*, *A. verna*, *A. ocreata*, *A. bisporigera*, *A. suballiacea*, *A. tenuifolia*, and *A. hygroscopica* [2,3]. Among the over 600 species of this genus that are poisonous to humans, *A. hemibapha* subsp. *javanica* is an edible mushroom. The fruiting bodies of *A. hemibapha* subsp. *javanica* are white before maturation but turn yellowish red with their caps turning from a convex to a flat shape as they mature. *A. hemibapha* subsp. *javanica* usually grows throughout summer to fall, and it is often confused with *A. subjunquillea* Imai, also known as the East Asian death cap, which is potentially fatal when ingested [2]. Although a number of studies have reported the biological activities of *A. hemibapha*, there are limited studies on *A. hemibapha* subsp. *Javanica.* A recent study demonstrated that *A. hemibapha* subsp. *javanica* is known to scavenge hydroxyl radicals [4]. *A. hemibapha* subsp. *javanica* also secretes mucilage polysaccharides which activate RAW264.7 cells to release nitric oxide and cytokine mitogen-activated protein kinase pathways, thereby enhancing immunity [4,5]. Nevertheless, to the best of our knowledge, *A. hemibapha* subsp. *javanica* has never been evaluated in terms of its chemical constituents.

In our continuous efforts to chemically study these uninvestigated Korean wild mushrooms as well as to discover novel bioactive compounds from natural sources [6,7,8], we conducted the first chemical analysis of *A. hemibapha* subsp. *javanica*, which resulted in the isolation of seven fatty acid derivatives (**1**–**7**) and three steroids (**8**–**10**) from the methanol (MeOH) extract of the fruiting bodies. These compounds were isolated using successive column chromatography and preparative and semi-preparative HPLC purification. The structures of the isolated compounds were determined using magnetic resonance (NMR) spectroscopy, physical data interpretation, and liquid chromatography-mass spectrometry (LC/MS) analyses. In the current study, the isolation of compounds **1**–**10**, their structural determination, and evaluation of their anti-*Helicobacter pylori* activity was also reported.

## 2. Results and Discussion

### 2.1. Isolation of Compounds

**Figure 1:**
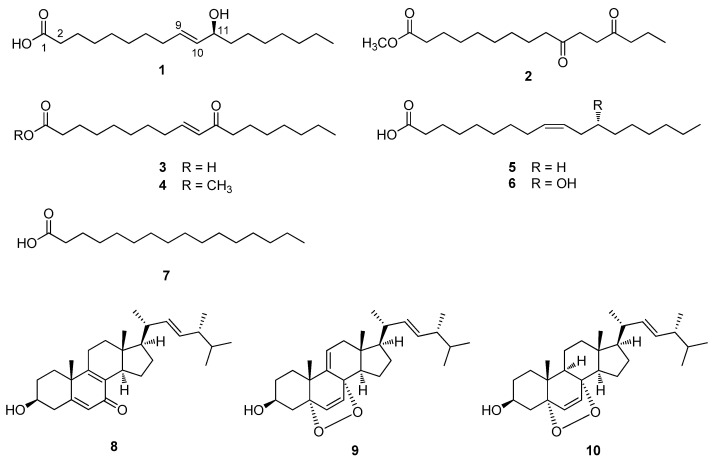
Chemical structure of compounds **1–10**.

The dried fruiting bodies of *A. hemibapha* subsp. *javanica* were extracted with 80% MeOH/H_2_O, which provided the resultant MeOH extract after rotary evaporation. The MeOH extract was solvent-partitioned with hexane, dichloromethane, ethyl acetate, and *n*-butanol to obtain four major fractions according to the order of polarity (Figure S4). The LC/MS analysis of the four fractions and thin-layer chromatography (TLC) analysis revealed that the hexane and ethyl acetate-soluble fractions were promising for chemical analysis as we observed major peaks characteristic of fatty acid derivatives and sterols, which are major constituents in mushrooms, in the hexane and ethyl acetate-soluble fractions. The chemical composition of *A. hemibapha* subsp. *javanica* was identified by intensive chemical analysis of the hexane and ethyl acetate-soluble fractions using successive column chromatography and preparative and semi-preparative HPLC purification (Figure S4). During the isolation procedure, the subfractions and isolated compounds were monitored by LC/MS, and seven fatty acid derivatives (**1**–**7**) and three steroids (**8**–**10**) were isolated (Figure 1).

### 2.2. Determination of the Structure of Compounds

**Figure 2:**
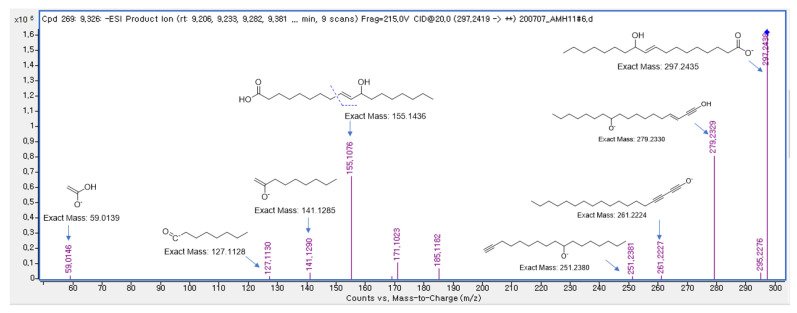
MS/MS data (MS^2^ 297.2 [M − H]^−^→full-scan) and fragmentation pathways of compound **1**.

Compound **1** was isolated as an amorphous gum. The molecular formula was established as C_18_H_34_O_3_ from the molecular ion peak [M − H]^−^ at *m/z* 297.2427 (calculated for C_18_H_33_O_3_, 297.2430) in the negative-ion mode of HR-ESIMS (Figure S1). The IR spectrum showed an absorption band of the hydroxyl group (3331 cm^−1^). The ^1^H NMR spectrum of compound **1** (Figure S2) showed signals of an olefinic pair at *δ*_H_ 5.59 (1H, dt, *J* = 15.5, 7.0 Hz) and 5.40 (1H, dd, *J* = 15.5, 7.0 Hz), oxygenated methine at *δ*_H_ 3.94 (1H, q, *J* = 7.0 Hz), terminal methyl group at *δ*_H_ 0.89 (3H, t, *J* = 7.0 Hz), and deshielded methylenes at *δ*_H_ 2.24 (2H, t, *J* = 7.5 Hz) and 2.03 (2H, m), and the overlapping signals are attributed to the remaining methylenes from 1.24 to 1.59 ppm. The coupling pattern of one of the olefinic protons [*δ*_H_ 5.40 (dd, *J* = 15.5, 7.0 Hz)] indicated that the oxygenated methine could be located next to the olefinic proton, and the olefinic protons were determined to have a *trans*-form. The overall ^1^H NMR data showed that the Compound **1** was a fatty acid derivative [9]. The double bond was located at C-9/C-10, as shown by the MS/MS data and fragmentation pathways, where the MS^2^ of compound **1** yielded main fragment ions at *m/z* 297.2 [M − H]^−^ and 155.1 [C_10_H_19_O]^−^ (Figure 2). Thus, the structure of compound **1** was determined to be (*E*)-11-hydroxy-9-octadecenoic acid, which has been reported previously without determining its absolute configuration [10].

**Figure 3:**
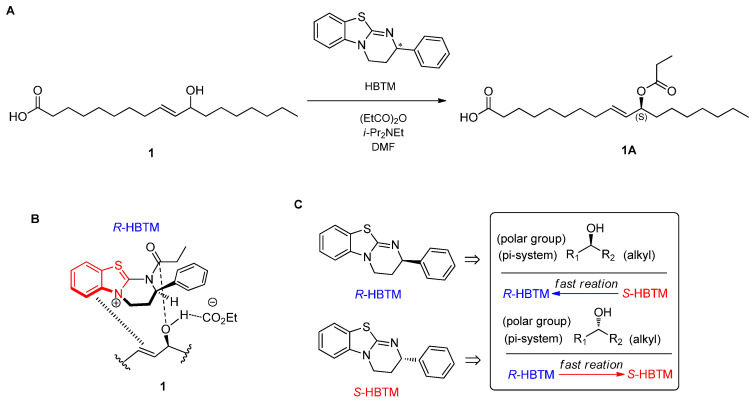
(**A**) CEA reaction for determining the absolute configuration of compound **1**. (**B**) Proposed favorable transition state of compound **1** in the reaction. (**C**) Mnemonic to predict the configuration of secondary alcohols in the CEA reaction.

To assign the absolute configuration of C-11 in compound **1**, a recently developed chemical-derivative method, competing enantioselective acylation (CEA) coupled with LC/MS analysis [11], was applied. The method uses homobenzotetramisole (HBTM) catalysts, in which the reaction rates of the parallel reactions are compared using LC/MS. For each parallel acylation reaction, two sets of compound **1** (each 0.2 mg) and *S*- and *R*-HBTM catalysts (each 10 μL) were reacted. Samples of each reaction were quantitatively analyzed using LC/MS to measure the reaction rate catalyzed by *S*- and *R*-HBTM. The acylated derivative (1A, [M − H]^−^ peak at *m/z* 353), esterified by propionic anhydride at the hydroxyl group of C-11, was expected because of the CEA reaction (Figure 3). The anticipated derivatives could be directly detected through the extracted ion chromatogram (EIC) of the LC/MS, where the peak areas of the acylated derivatives were compared (Figure S3) in samples of both parallel reactions to determine the reaction rate. The results revealed that the esterification reaction with *R*-HBTM was faster than that with *S*-HBTM (Figure 3), suggesting that compound **1** has an 11*S*-configuration, according to the Mnemonic to predict the configuration of secondary alcohols in the CEA reaction (Figure 3) [11]. Collectively, the complete structure of compound **1** was determined to be (9*E*,11*S*)-hydroxyoctadecenoic acid (Figure 1) and it was termed as amanitahemic acid A.

The other isolated compounds were identified as methyl ester 10,13-dioxo-hexadecanoic acid (2) [12], (9*E*)-11-oxo-9-octadecenoic acid (3) [13], (9*E*)-methyl ester 9-octadecenoic acid (4) [14], oleic acid (5) [15], ricinoleic acid (6) [16], palmitic acid (7) [16], (3*β*,22*E*)-3-hydroxyergosta-5,8,22-trien-7-one (8) [17], 9,11-dehydroergosterol peroxide (9) [18], and ergosterol peroxide (10) [19] (Figure 1). These were identified by comparing their NMR spectroscopic and physical data with those previously reported, along with the data from LC/MS (Table S2).

### 2.3. Evaluation of Antibacterial Activity of the Isolated Compounds against H. pylori

*Helicobacter pylori* is a major public health issue worldwide, affecting approximately 50% of the global population [20]. Eradication of *H. pylori* helps treat both gastritis and gastric ulcers, and even gastric cancer because the presence of *H. pylori* was associated with the gastric pathologies [21]. However, clinical failures due to antibiotic resistance are of increasing concern [22,23]. Thus, there is an urgent need to develop novel antibiotics against *H. pylori*. Prior to the test for the isolated compounds, the anti-*H. pylori* activity of the MeOH extract and solvent-partitioned fractions was evaluated using a clinical strain of *H. pylori* 51. Although the MeOH extract showed no inhibitory activity against *H. pylori* strain 51, hexane fractions showed weak inhibitory activity (Table S3). Next, the isolated compounds **1**–**10** were evaluated for antibacterial activity against *H. pylori* strain 51 (Table 1). Among the isolates, compound **3** exhibited antibacterial activity against *H. pylori* strain 51 with 38.0% inhibition, comparable to that of quercetin (34.4% inhibition) as a positive control. Specifically, compound **4** displayed the most potent antibacterial activity against *H. pylori* strain 51 with 80.5% inhibition at the final concentration of 100 μm (Table 1), and it showed a MIC_50_ value of 72 μm. The other compounds failed to show anti-*H. pylori* activity. Based on these findings, it was found that the presence of an α,β-unsaturated carbonyl moiety in fatty acid derivatives can be important for the anti-*H. pylori* activity and the hydroxyl group may decrease the inhibitory activity. In fact, it has been known that *H. pylori* produces a potent urease, which catalyzes the hydrolysis of urea to produce ammonia for neutralizing the acidic condition of stomach, and simple α,β-unsaturated ketones inhibit urease activity by binding to the cysteinyl residue in the active sites of the enzyme [24]. Further study is required to elucidate the exact mechanism of compounds **3** and **4** to inhibit the growth of *H. pylori*.

## 3. Materials and Methods

### 3.1. General Experimental Procedure

The information on general experimental procedure is provided in Supplementary Materials.

### 3.2. Fungal Material

The fruiting bodies of *A. hemibapha* subsp. *javanica* were collected from Yunggeolleung, Hwaseong, GyeongGi-do, Korea, in August 2015. The samples were identified by one of the authors (R. Ryoo). The DNA identification of this material was confirmed by the modified method of Lee and Taylor [25]. The nuclear ribosomal internal transcribed spacer (ITS) region of the DNA sequence was amplified by the fungal-specific PCR primers ITS1 and ITS4 [26]. This sequence was matched with *A. hemibapha* subsp. *javanica* with the highest score searched in NCBI BLAST database. A voucher specimen (SKKU 2015-08-AH) was deposited in the herbarium of the School of Pharmacy, Sungkyunkwan University, Suwon, Korea.

### 3.3. Extraction and Separation/Isolation

The dried fruiting bodies of *A. hemibapha* subsp. *javanica* (1.1 kg) were extracted using 80% aqueous MeOH thrice (3 L × 24 h each) at room temperature. Extracts were filtered, and the filtrate was evaporated under reduced pressure using a rotary evaporator to obtain a crude MeOH extract (8.6 g). The extract was suspended in distilled water (700 mL) and MeOH (30 mL) and successively solvent-partitioned with hexane (HX), dichloromethane (CH_2_Cl_2_), ethyl acetate (EA), and *n*-butanol, yielding soluble fractions of hexane (1.5 g), CH_2_Cl_2_ (381.7 mg), EtOAc (305.7 mg), and *n*-butanol (1.3 g). The HX fraction (1.5 g) was subjected to silica gel column chromatography (CC) (hexane/EtOAc, from 30:1 to 1:1) to obtain 10 fractions (Fr. A1–A10). Fraction A7 and fraction A8 were combined because of their similar major spots on TLC (Merck, Darmstadt, Germany) analysis, run at hexane/EtOAc (3:1) and MeOH/H_2_O (9:1) on silica and reverse-phase TLC plates, respectively. The combined fraction (160.8 mg) was fractionated using preparative HPLC (Shimadzu, Tokyo, Japan) (MeOH/H_2_O, from 83:17 to 100:0) on an Agilent Eclipse C_18_ column (Agilent Technologies, Santa Clara, CA, USA) (250 × 21.2 mm, 5 μm; flow rate: 5 mL/min; elution time: 82.0 min), yielding six subfractions (Fr. A71–A76). Fraction A74 (27.1 mg) was purified using semi-preparative HPLC (MeOH/H_2_O, 80:20) employing a Phenomenex Luna phenyl-hexyl column (Phenomenex, Torrance, CA, USA) (250 × 10 mm i.d., flow rate: 2 mL/min; elution time: 72.0 min) to yield compounds **2** (*t_R_* 37.0 min, 2.1 mg) and **4** (*t_R_* 53.5 min, 1.5 mg) (Table S1). Fraction A9 and fraction A10 were also combined based on TLC analysis results, run at hexane/EtOAc (3:1) and MeOH/H_2_O (9:1) on silica and reverse-phase TLC plates, respectively, and the combined fraction (184.7 mg) was fractionated using preparative HPLC (MeOH/H_2_O, from 80:20 to 100:0) with an Agilent Eclipse C_18_ column (Agilent Technologies) (250 × 21.2 mm, 5 μm; flow rate: 5 mL/min; elution time: 82.0 min), which yielded five subfractions (Fr. A91–A95). Fraction A93 (26.8 mg) was purified using semi-preparative HPLC (MeCN/H_2_O, 58:42) on a Phenomenex Luna phenyl-hexyl column (Phenomenex) (250 × 10 mm i.d., flow rate: 2 mL/min; elution time: 62.0 min) and yielded compounds **6** (*t_R_* 25.0 min, 2.5 mg), **1** (*t_R_* 27.0 min, 1.8 mg), and **3** (*t_R_* 36.0 min, 1.4 mg) (Table S1). Fraction A95 (40.4 mg) was also purified using semi-preparative HPLC (MeCN/H_2_O, 68:32) with a Phenomenex Luna phenyl-hexyl column (Phenomenex) (250 × 10 mm i.d., flow rate: 2 mL/min; elution time: 82.0 min) and yielded compounds **8** (*t_R_* 40.5 min, 0.5 mg), **9** (*t_R_* 51.0 min, 0.7 mg), and **10** (*t_R_* 53.5 min, 1.9 mg) (Table S1). The EA fraction (305.7 mg) was fractionated using preparative HPLC (MeOH/H_2_O, from 30:70 to 100:0) employing an Agilent Eclipse C_18_ column (Agilent Technologies) (250 × 21.2 mm, 5 μm; flow rate: 5 mL/min; elution time: 82.0 min), which yielded five subfractions (Fr. B1–B5). Fraction B5 (59.2 mg) was purified using semi-preparative HPLC (MeCN/H_2_O, 68:32) with a Phenomenex Luna phenyl-hexyl column (Phenomenex) (250 × 10 mm i.d., flow rate: 2 mL/min; elution time: 82.0 min) and yielded compounds **7** (*t_R_* 38.0 min, 1.7 mg) and **5** (*t_R_* 43.0 min, 4.7 mg) (Table S1).

#### Amanitahemic Acid A (**1**)

Amorphous gum; [α]25D -7.7 (*c* 0.05, MeOH); UV (MeOH) *λ*_max_ (log *ε*) = 215 (2.6) nm; IR (KBr) *ν*_max_: 3331, 3178, 3015, 1625, 1018 cm^−1^; (–)-high-resolution electrospray ionization mass spectroscopy (HR-ESIMS) *m/z* 297.2427 [M − H]^−^ (calculated for C_18_H_33_O_3_, 297.2430); ^1^H NMR (800 MHz, CD_3_OD): *δ* 5.59 (1H, dt, *J* = 15.5, 7.0 Hz, H-12), 5.40 (1H, dd, *J* = 15.5, 7.0 Hz, H-13), 3.94 (1H, q, *J* = 7.0 Hz, H-14), 2.24 (2H, t, *J* = 7.5 Hz, H_2_-2), 2.03 (2H, m, H_2_-11), 1.59 (2H, m), 1.51 (2H, m), 1.24-1.44 (18H, m), 0.89 (3H, t, *J* = 7.0 Hz, H_3_-18) (Figure S2).

### 3.4. MS/MS Analysis of Compound ***1***

Stock solution of compound **1** was prepared by dissolving 0.1 mg of sample in 200 μL MeOH. The solution was further diluted with MeOH, filtered through a 0.45 μm hydrophobic PTFE filter, and analyzed using LC/MS/MS, Agilent 1290 Infinity II series with a 6545 LC/Q-TOF mass spectrometer (Agilent Technologies, Santa Clara, CA, USA). The analysis was conducted by injecting 1 μL of the sample using an Agilent Eclipse Plus C_18_ RRHD (1.8 μm, 2.1 × 50 mm) set at 30 °C. The mobile phase consisting of formic acid in H_2_O (0.1% (*v*/*v*)) (A) and formic acid in acetonitrile (0.1% (*v*/*v*)) (B) was delivered at a flow rate of 0.3 mL/min by applying the following programmed gradient elution: 0–3.0 min, 10% (B); 3.0–10.0 min, 10–100% (B); 10.0–12.0 min, 100% (B); 12.0–15.0 min, 10% (B). The MS system was equipped with an ESI source and operated in both negative and positive ionization modes with a data acquisition range from *m/z* 100 to 600.

### 3.5. Experimental Procedures to Determine the Absolute Configuration of Compound ***1***

#### 3.5.1. CEA Reaction

Parallel reactions for the CEA reaction were performed as reported by Lee et al. [11], using *S*- and *R*-HBTM (Sigma-Aldrich, Burlington, MA, USA). Compound **1** (0.2 mg, 0.67 μmol) was transferred to two transparent and capped 5 mL vials at room temperature, and DMF (90 μL) was added as the organic solvent for the CEA reaction. Both *S*- and *R*-HBTM (10 μL, 0.38 μmol) were added, and *N*,*N*-diisopropylethylamine (1.0 μL, 5.3 μmol) was successively transferred. Propionic anhydride (0.6 μL, 5.3 μmol) was added to start the CEA reaction. After 10 min, 2 μL aliquots from each reaction were acquired for LC/MS analysis and quenched with 98 μL of MeOH to obtain a total volume of 100 μL.

#### 3.5.2. LC/MS Analysis

An aliquot (5 μL) of the sample (100 μL) acquired from each parallel reaction was directly injected into the LC/MS and analyzed using an analytical Kinetex C18 100 Å column (Phenomenex) (C_18_, 4.6 × 100 mm, 3.5 μm, flow rate: 0.3 mL/min), and full scans in positive- and negative-ion modes (scan range *m/z* 100−1000) were applied to identify the desired acylated derivative. The mobile phase consisted of 0.1% (*v*/*v*) formic acid in distilled water (A) or acetonitrile (B) with a gradient solvent system as follows: 10%−100% B for 10 min, 100% B (isocratic) for 5 min, and then 10% B (isocratic) for 5 min for the post-run washing procedure of the column. The reaction rate catalyzed by both *S*- and *R*-HBTM was determined by measuring the peak areas of the acylated derivatives.

### 3.6. H. pylori Culture

A clinical strain of *H. pylori* 51 was provided by the *H. pylori* Korean Type Culture Collection, School of Medicine, Gyeongsang National University, Korea. The strain was grown and maintained on Brucella agar medium (BD Co., Sparks, MD, USA) supplemented with 10% horse serum (Gibco, New York, NY, USA). The culture conditions were 37 °C, 100% humidity, and 10% CO_2_ for 2–3 days.

### 3.7. Anti-H. pylori Activity

Minimal inhibitory concentrations (MICs) were determined by broth dilution method previously reported [27]. Twenty microliters of bacterial colony suspension equivalent to 2–3 × 10^8^ cfu/mL and twenty microliters of two-fold diluted samples and controls were added to each well of a 6-well plate containing Brucella broth medium (BD Co., Sparks, MD, USA) supplemented with 10% horse serum. The final volume was brought to 2 mL. After 24 h of incubation, bacterial growth was evaluated by measuring the optical density at 600 nm on spectrophotometer (Optizen POP, Mecasys, Daejeon, Korea). MIC_50_ and MIC_90_ values were defined as the lowest concentrations of samples at which bacterial growth was inhibited by 50% and 90%, respectively, and were calculated using GraphPad Version 5.01 (GraphPad Software, Inc., San Diego, CA, USA). All of the values were obtained from three independent experiments.

## 4. Conclusions

In this study, the first chemical investigation of the fruiting bodies of *A. hemibapha* subsp. *javanica*, an edible mushroom among *Amanita* species, led to the isolation and identification of seven fatty acid derivatives (**1**–**7**) and three steroids (**8**–**10**). The structure of compound **1** (amanitahemic acid A), including its absolute configuration, was elucidated using ^1^H NMR, MS/MS fragmentation data, and the application of CEA reaction coupled with LC/MS. In the anti-*H. pylori* activity test, compound **3** showed antibacterial activity against *H. pylori* strain 51 with 38.0% inhibition, comparable to that of quercetin (34.4% inhibition). Specifically, compound **4** displayed the most potent antibacterial activity against *H. pylori* strain 51 with 80.5% inhibition at the final concentration of 100 μm with the MIC_50_ value of 72 μm. Based on these findings, we conclude that compound **4** could be used to develop novel antibiotics against *H. pylori*.

## Figures and Tables

**Table 1:** Anti-*H. pylori* activity of compounds **1**–**10**.

Compound	Concentration (μm)	Inhibition (%)	MIC_50_ (μm)	MIC_90_ (μm)
**1**	100	27.4		
**2**		3.4		
**3**		38.0		
**4**		80.5	72	>100
**5**		14.6		
**6**		15.8		
**7**		0.0		
**8**		5.7		
**9**		0.6		
**10**		4.9		
Quercetin ^a^	100	34.4		
Metronidazole ^a^		97.0	17	46

^a^ Positive controls.

## Data Availability

Data is contained within the article and supplementary materials.

## Supplementary Materials

The following are available online at https://www.mdpi.com/article/10.3390/ph15020152/s1; Figure S1: HR-ESIMS data of **1**; Figure S2: ^1^H NMR spectrum of **1** (CD_3_OD, 800 MHz); Figure S3: EIC of LC/MS data of acylated derivative from CEA reaction of **1**; Figure S4: The separation scheme of compounds **1**–**10**; Table S1: HPLC chromatogram for the identification of compounds **1**–**10**; Table S2: LC-MS identification of compounds **1**–**10**; General experimental procedures; Table S3: Anti-*H. pyroli* activity of the MeOH extract and fractions derived from the solvent partitioning.
