# Supplementary material for: First Chemical Investigation of Korean Wild Mushroom, Amanita hemibapha subsp. javanica and the Identification of Anti-Helicobacter pylori Compounds
Source: Pharmaceuticals (Basel). 2022 Jan 27;15(2):152. doi: 10.3390/ph15020152 (PMC8874524; doi:10.3390/ph15020152)
Supplement: Supplementary file 1 [file pharmaceuticals-15-00152-s001.zip › pharmaceuticals-1530658-supplementary.pdf]

Supplementary Materials

First Chemical Investigation of Korean Wild Mushroom, *Amanita hemibapha* subsp. *javanica* and the Identification of Anti-*Helicobacter pylori* Compounds

Seulah Lee [1,2], Akida Alishir [1], Tae Wan Kim [1], Dong-Min Kang [3], Rhim Ryoo [4], Changhyun Pang [5], Mi-Jeong Ahn [3], and Ki Hyun Kim [1,*]

[1] School of Pharmacy, Sungkyunkwan University, Suwon 16419, Korea; seulah@kopri.re.kr (S.L.); akida.alishir@gmail.com (A.A.); asde8282@naver.com (T.W.K.)

[2] Division of Life Sciences, Korea Polar Research Institute, KIOST, Incheon, 21990, Korea

[3] College of Pharmacy and Research Institute of Pharmaceutical Sciences, Gyeongsang National University, Jinju 52828, Korea; kdm7105@gnu.ac.kr (D.M.K.); amj5812@gnu.ac.kr (M.J.A.)

[4] Special Forest Products Division, Forest Bioresources Department, National Institute of Forest Science, Suwon 16631, Korea; rryoo@korea.kr (R.R.)

[5] School of Chemical Engineering, Sungkyunkwan University, Suwon 16419, Korea; chpang@skku.edu (C.P.)

***** Correspondence: khkim83@skku.edu (K.H.K.); +82-31-290-7700 (K.H.K.)

List of Supplementary Materials

Figure S1. HR-ESIMS data of **1**……………………………………………………………..……………………………………………..…3

Figure S2. ^{1}H NMR spectrum of **1** (CD_3OD, 800 MHz)……………………………..………………………………………………4

Figure S3. EIC of LC/MS data of acylated derivative from CEA reaction of **1**………………………………………………………………5

Table S1. HPLC chromatogram for the identification of compounds **1-10** ……………………………………………………………………..6

Table S2. LC-MS identification of compounds **1-10** ……………………………………………………………………………..…………..7

General experimental procedures …………………………………………………………..……………………………………………8

Figure S4. The separation scheme of compounds **1–10** ……………………………………………………………………………………9

Table S3. Anti-*H. pyroli* activity of the MeOH extract and fractions derived from the solvent partitioning……………………………………10

Figure S1. HR-ESIMS data of **1**

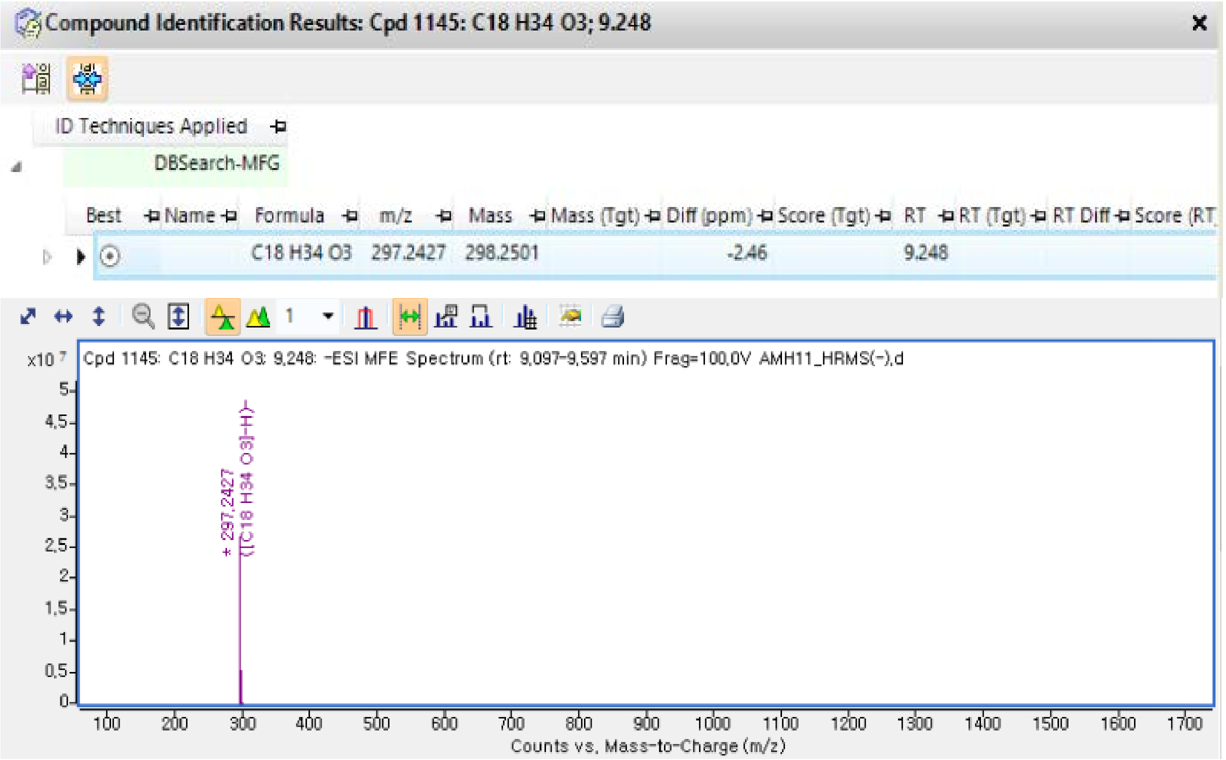

Figure S2. ^{1}H NMR spectrum of **1** (CD_3OD, 800 MHz)

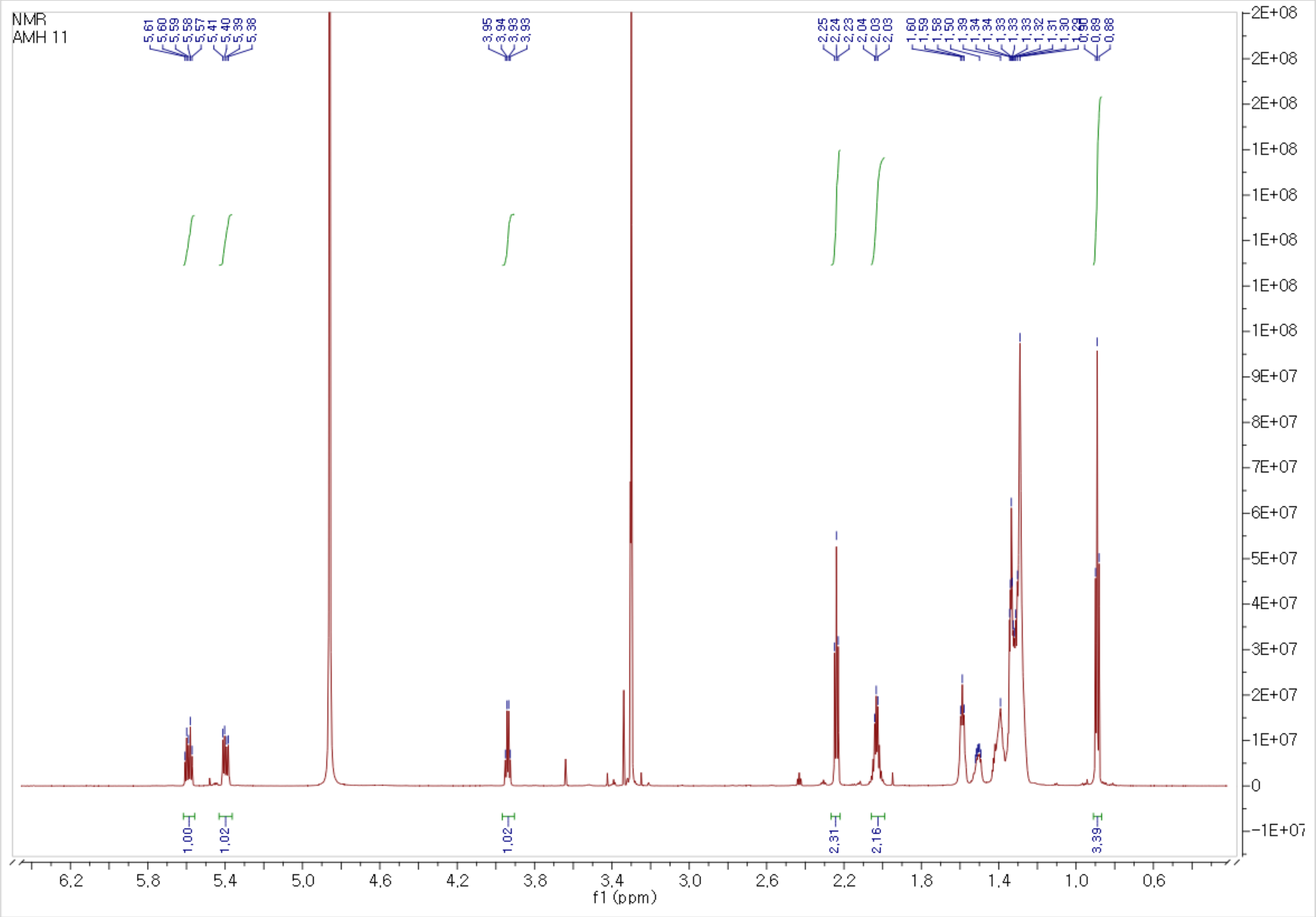

Figure S3. EIC of LC/MS data of acylated derivative from CEA reaction of **1**: (A) An acylated derivative of compound **1** in *S*-HBTM catalyzed acylation reaction; (B) An acylated derivative of compound **1** in *R*-HBTM catalyzed acylation reaction.

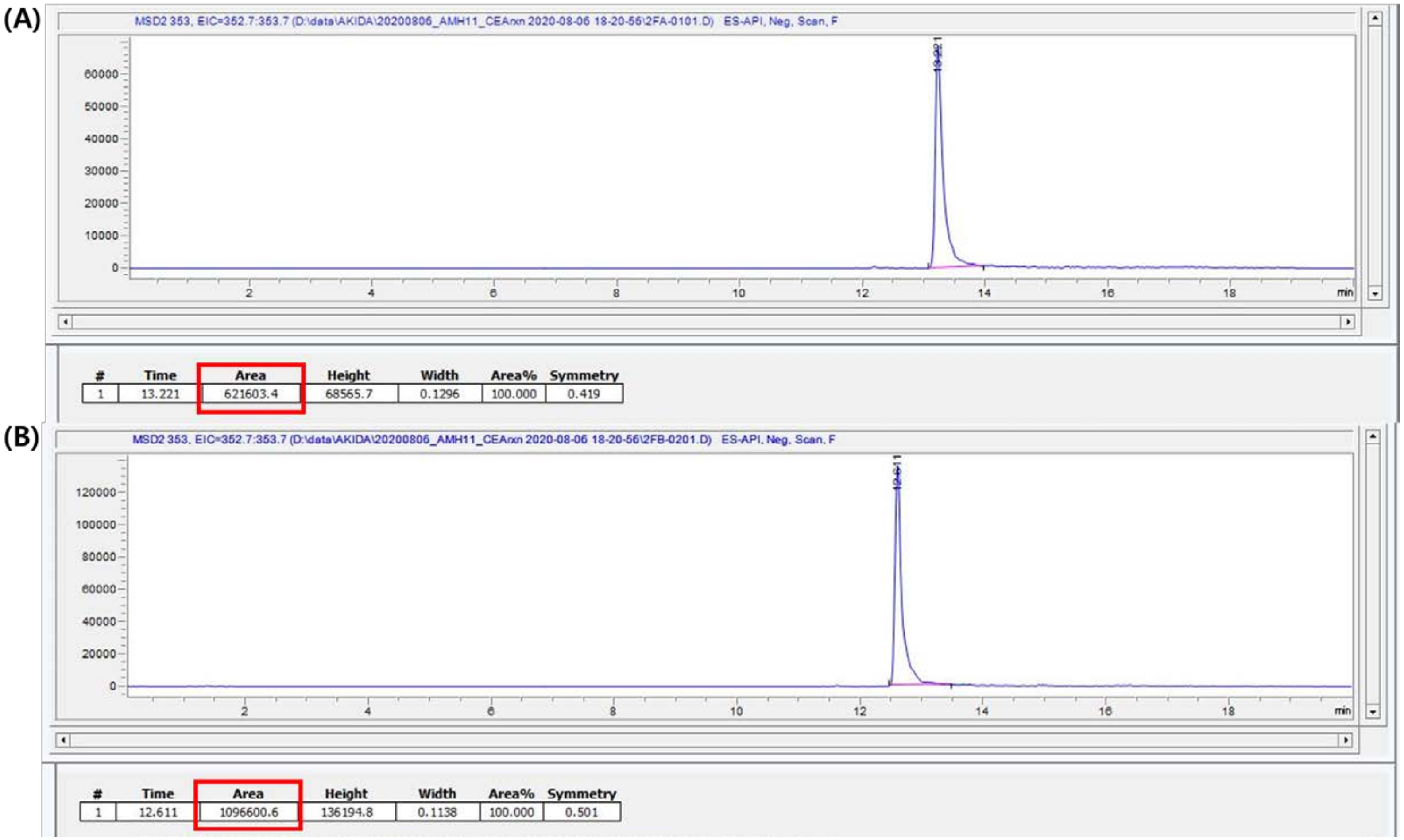

Table S1. HPLC chromatogram for the identification of compounds **1-10**.

HPLC chromatograms	Solvent conditions	Elution time
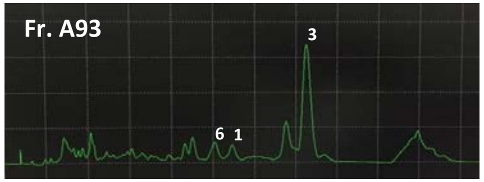	MeCN/H_2O, 58:42	62.0 min
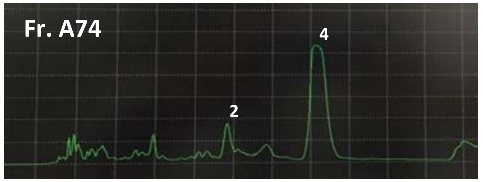	MeOH/H_2O, 80:20	72.0 min
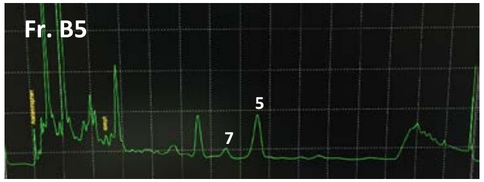	MeCN/H_2O, 68:32	82.0 min
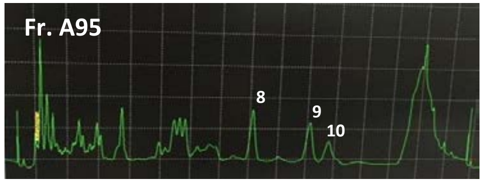	MeCN/H_2O, 68:32	82.0 min

Table S2. LC-MS identification of compounds **1-10**

Compounds	Retention time (min)	Maximum wavelength (nm)	Solvent elution condition for LC/MS formic acid in H_2O [0.1% (v/v)] (A) and formic acid in MeOH (B)	Molecular ion (M^+)	Fragmentation ions
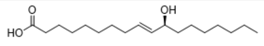 Amanitahemic acid A (**1**)	6.95	204	gradient elution: 0–10.0 min, 70%-100% (B); 10.0–15.0 min, 100% (B); 15.0–16.0 min, 100-70% (B); 16.0–20.0 min, 70% (B)	298	59, 127, 141, 155, 251, 261, 279, 297
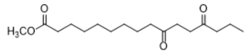 Methyl ester 10,13-dioxo-hexadecanoic acid (**2**)	8.73	202	gradient elution: 0–10.0 min, 70%-100% (B); 10.0–15.0 min, 100% (B); 15.0–16.0 min, 100-70% (B); 16.0–20.0 min, 70% (B)	298	244, 280
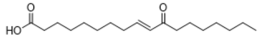 (9*E*)-11-Oxo-9-octadecenoic acid (**3**)	7.21	228	gradient elution: 0–10.0 min, 70%-100% (B); 10.0–15.0 min, 100% (B); 15.0–16.0 min, 100-70% (B); 16.0–20.0 min, 70% (B)	296	169, 251, 279
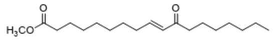 (9*E*)-Methyl ester 9-octadecenoic acid (**4**)	8.86	229	gradient elution: 0–10.0 min, 70%-100% (B); 10.0–15.0 min, 100% (B); 15.0–16.0 min, 100-70% (B); 16.0–20.0 min, 70% (B)	310	153, 211
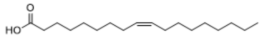 Oleic acid (**5**)	11.24	202	gradient elution: 0–10.0 min, 70%-100% (B); 10.0–15.0 min, 100% (B); 15.0–16.0 min, 100-70% (B); 16.0–20.0 min, 70% (B)	282	55, 74, 97, 137, 180, 222, 264
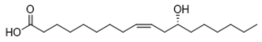 Ricinoleic acid (**6**)	7.43	202	gradient elution: 0–10.0 min, 70%-100% (B); 10.0–15.0 min, 100% (B); 15.0–16.0 min, 100-70% (B); 16.0–20.0 min, 70% (B)	298	83, 97, 111, 185, 263, 281
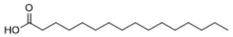 Palmitic acid (**7**)	10.96	202	gradient elution: 0–10.0 min, 70%-100% (B); 10.0–15.0 min, 100% (B); 15.0–16.0 min, 100-70% (B); 16.0–20.0 min, 70% (B)	256	43, 73, 117, 129, 213
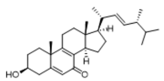 (3*β*,22*E*)-3-Hydroxyergosta-5,8,22-trien-7-one (**8**)	11.08	250	gradient elution: 0–10.0 min, 70%-100% (B); 10.0–15.0 min, 100% (B); 15.0–16.0 min, 100-70% (B); 16.0–20.0 min, 70% (B)	410	284
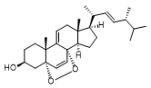 9,11-Dehydroergosterol peroxide (**9**)	11.10	205	gradient elution: 0–10.0 min, 70%-100% (B); 10.0–15.0 min, 100% (B); 15.0–16.0 min, 100-70% (B); 16.0–20.0 min, 70% (B)	426	189, 303, 339, 356, 367, 409
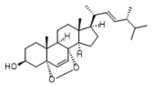 Ergosterol peroxide (**10**)	11.53	203	gradient elution: 0–10.0 min, 70%-100% (B); 10.0–15.0 min, 100% (B); 15.0–16.0 min, 100-70% (B); 16.0–20.0 min, 70% (B)	428	191, 305, 341, 358, 369, 411

General experimental procedures

Optical rotations were measured on a Jasco P-1020 polarimeter (Jasco, Easton, MD, USA). Infrared (IR) spectra were recorded on a Bruker IFS-66/S FT-IR spectrometer (Bruker, Karlsruhe, Germany). Ultraviolet (UV) spectra were acquired on an Agilent 8453 UV-visible spectrophotometer (Agilent Technologies, Santa Clara, CA). High-resolution (HR)-electrospray ionization (ESI) mass and tandem mass (MS/MS) spectra were recorded on an Agilent 1290 Infinity II series with 6545 LC/Q-TOF mass spectrometer (Agilent Technologies). NMR spectra were measured using a Bruker AVANCE III (Bruker). Preparative high-performance liquid chromatography (HPLC) was conducted using a Waters 1525 binary HPLC pump with Waters 996 photodiode array detector (Waters) and an Agilent Eclipse C_{18} column (250 × 21.2 mm, 5 μm; flow rate: 5 mL/min) (Agilent Technologies), and semi-preparative HPLC used a Shimadzu Prominence HPLC System with SPD-20A/20AV Series Prominence HPLC UV-Vis Detectors (Shimadzu, Tokyo, Japan). LC/MS analysis was performed on an Agilent 1200 series HPLC system with a diode array detector and 6130 Series ESI mass spectrometer using an analytical Kinetex C18 100 Å column (100 mm × 2.1 mm i.d., 5 μm) (Phenomenex, Torrance, CA). Column chromatography used silica gel 60, 230–400 mesh (Merck, Darmstadt, Germany). Thin-layer chromatography (TLC) was conducted using precoated silica gel F_{254} plates and reverse-phase (RP)-18 F_{254s} plates (Merck). Spots on TLC were detected using UV and heating after dipping in anisaldehyde-sulfuric acid.

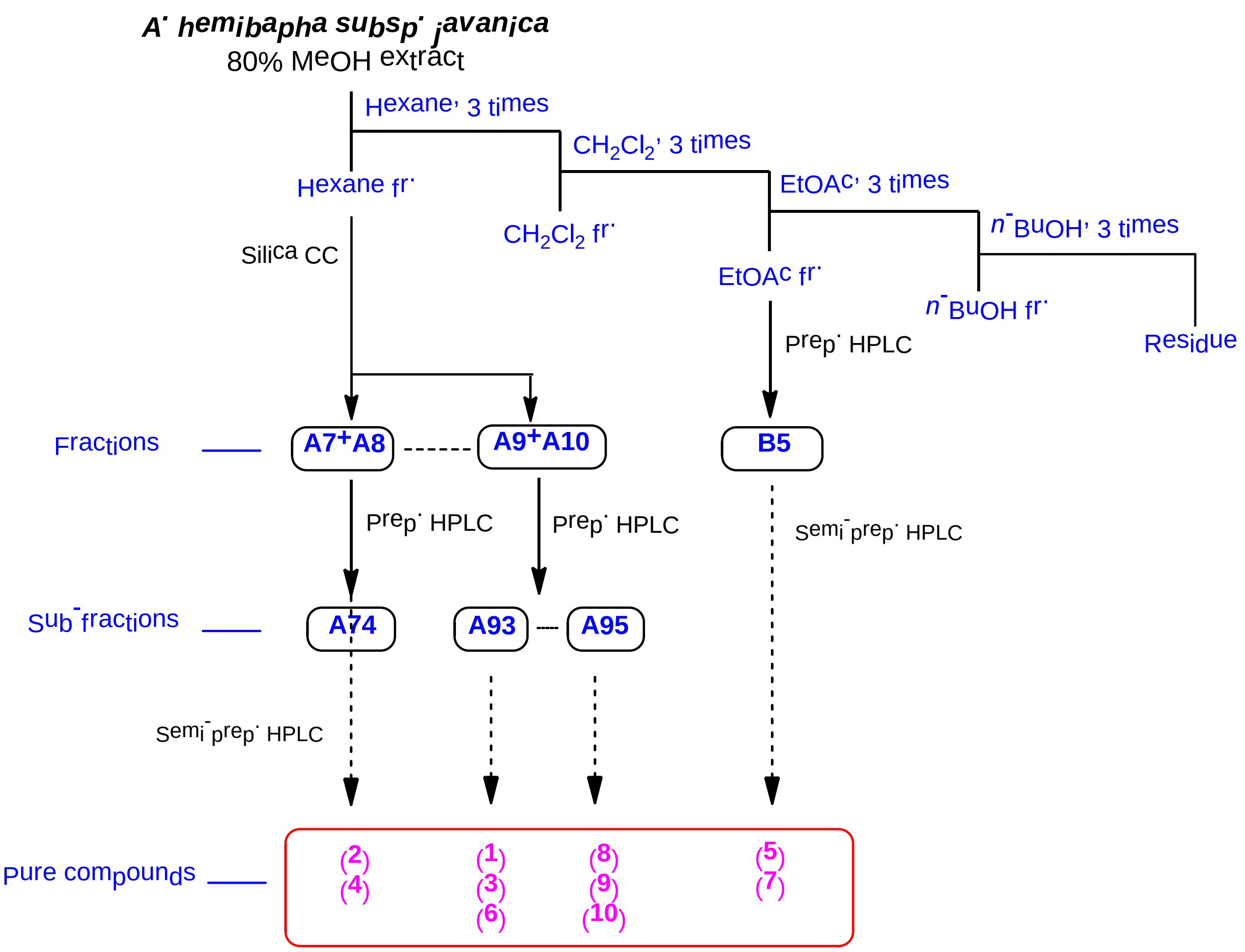

Figure S4. The separation scheme of compounds **1–10**.

Table S3. Anti-*H. pyroli* activity of the MeOH extract and fractions derived from the solvent partitioning.

**Sample**	**Concentrations**	**Inhibition (%)**
MeOH extract	100 μg/mL	0.0
Hexane fraction		12.7
Dichloromethane fraction		7.1
Ethyl acetate fraction		5.9
n-BuOH fraction		2.0
